# Social psychological mechanisms and processes in a novel, health professional-led, self-management intervention for older stroke individuals: a synthesis and phenomenological study

**DOI:** 10.1186/s12913-019-4150-x

**Published:** 2019-05-22

**Authors:** Susanne Lillelund Sørensen, Sedsel Kristine Stage Pedersen, Hanne Pallesen

**Affiliations:** Hammel Neurorehabilitation Centre and University Research Clinic, RM, University of Aarhus, Voldbyvej 15, 8450, Hammel, Aarhus, Denmark

**Keywords:** Self-management, Stroke, Caregivers, Mentors, Social support

## Abstract

**Background:**

A good portion of stroke patients in Western countries are over 65 of age. Because of sequelae, they often lead more isolated lives after the stroke. In terms of social reintegration, this group of patients is especially vulnerable. Reintegration into the community post-stroke greatly depends on support from family. However, the stroke individual’s closest relatives are at risk of becoming overburdened.

The objectives are to describe the social psychological mechanisms and processes involved in a novel self-management intervention, and to evaluate their feasibility and acceptability from the stroke individuals’, the informal caregivers’ and the mentors’ perspectives, before implementation into a randomised controlled trial.

**Methods:**

Qualitative interviews were conducted and analysed using a phenomenological approach. Informants comprised four stroke individuals, three informal caregivers and two mentors.

The UK Medical Research Council Framework for developing and evaluating complex interventions was used in the evaluation design of the intervention.

**Results:**

Six social psychological mechanisms were revealed as the mentors’ focus areas in their interaction with stroke individuals and informal caregivers: a) Tailored approach – by individual preferences, b) Dialogue-based communication, c) Development of a good relationship, d) Transfer of activities to everyday and social contexts, e) Involvement of relatives and social networks, and f) Supporting tools – to optimise actions and communication. Furthermore, interaction processes between the stroke individual and the informal caregiver and the mentors occurred, and generated processes of change and learning in the stroke individual and the informal caregiver. The mechanisms and processes described were perceived as feasible and acceptable to the informants – with the exception of the technological supporting tool.

**Conclusion:**

The social psychological mechanisms and processes involved in the intervention indicated a positive association to self-management behaviour from the informants’ perspectives. The informants evaluated them to be relevant and meaningful in the novel self-management intervention.

**Trial registration:**

ClinicalTrials.gov NCT03183960. Reg. June 15, 2017.

**Electronic supplementary material:**

The online version of this article (10.1186/s12913-019-4150-x) contains supplementary material, which is available to authorized users.

## Background

Worldwide, stroke is a major cause of disability, and the number of people living with the consequences is increasing globally [1]. In Denmark, about 93,000 people are living with post-stroke consequences in a population of 5.5 million [2]. About two-thirds of all new incidents of stroke – approximately 10,000 annually – strike older people over the age of 65 [2].

In Denmark, one can be considered to be an old-age pensioner and receive pensioners’ rights from the month one turns 65 [3]. The majority of people over the age of 65 receive a retirement pension, and are no longer part of the labour market [4, 5]. When work-based social relationships end, old-age pensioners are at risk of experiencing loneliness [6]. Old-age pensioners living with sequelae post-stroke constitute an especially vulnerable group of older people, in terms of social reintegration [6, 7]. One study found that stroke individuals lived a more isolated life five years post-stroke, with fewer social relations and less participation in the community, compared with their life before the stroke [8].

Reintegration into the community post-stroke depends to a great extent on support from the family [9, 10]. The closest family members often experience an increased level of burden. The main burden is often carried by the spouse, who becomes an informal caregiver [7, 10]. Informal caregiver are defined as any relative, partner, friend or neighbour who has a significant personal relationship with, and provides a broad range of assistance for, an older person or an adult with a chronic or disabling condition. These individuals may be primary or secondary caregivers and live with, or separately from, the person receiving care [11]. It has been shown that the stroke individual’s closest relatives are at risk of developing anxiety and depression [7]. Because stroke affects the whole family, especially the spouse, stroke rehabilitation must focus not only on supporting stroke individuals to rebuild their former, or create new, social networks, but also on reducing the burden on informal caregivers [7].

A stroke can have an impact on multiple levels – physical, psychological, cognitive and behavioural – and can cause an unexpected life-course disruption [12, 13]. This could lead to reduced self-management – understood as an individual’s ability to manage symptoms, treatment, physical and psychological consequences, and lifestyle changes inherent in living with chronic disease [14]. In this definition, self-management focuses on those actions individuals use directly to manage their lives with a long-term condition and to maintain the best possible quality of life [15–17]. Evidence suggests that it is worthwhile supporting self-management in people with chronic conditions, in particular by focusing on behaviour change and by supporting self-efficacy [15]. Self-management support is the assistance professionals or informal caregivers provide to encourage a person to improve health-related behaviours in everyday life [15, 18].

The current study is a preliminary part of the larger project *‘Stroke - 65 plus. Continued Active Life’* [19], which aims to support older stroke individuals and their informal caregivers to maintain their former active lifestyle, or to create a new, meaningful life perspective. This paper describes the evaluation of the mechanisms and processes in a novel, health professional-led, self-management support intervention.

**Fig. 1:**
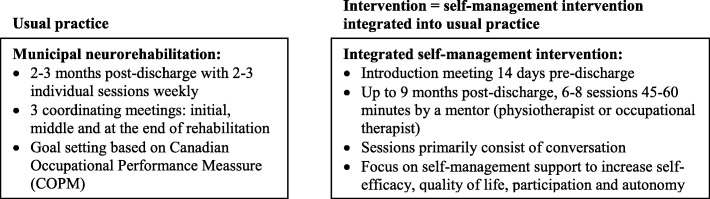
Description of overall framework in usual practice compared to the integrated self-management intervention

The larger randomised controlled trial (RCT) *‘Stroke - 65 plus. Continued Active Life’* has been described in detail elsewhere [19]. In the RCT, stroke individuals over the age of 65 will be randomised to either conventional neurorehabilitation or to an integrated, health professional-led, self-management support intervention. Those randomised to the intervention, which is integrated into usual practice, will receive behaviourally-focused self-management support designed to increase self-management, self-efficacy, quality of life, participation and autonomy. The intervention begins approximately two weeks before discharge from hospital. Post-discharge, the stroke individuals will be offered six to eight sessions of 45–60 min duration by a physiotherapist or an occupational therapist – who, from hereon in the paper, will be referred to as mentors. Usual practice versus the integrated self-management intervention is shown in Fig. 1.

In accordance with UK Medical Research Council (MRC) guidance, the self-management intervention is characterized as a complex intervention [20], due to complexity in not only the interacting processes, but also the behavioural patterns and the target outcomes.

The focus of this study is to unravel the mechanisms and processes in the intervention and examine how they relate to each other and whether they are feasible and acceptable to the stroke individuals and the informal caregivers. This is in order to make a transparent description that will be implementable in the larger project.

Evidence suggests that self-management programmes designed especially to encourage people to take an active part in the management of their own situation might be beneficial to people living in the community after a stroke [21, 22]. A recently published review showed that stroke individuals might improve their quality of life and self-efficacy by taking part in such programmes [22].

Two RCTs focusing on, respectively, a community-based rehabilitation programme and a self-management programme for middle-aged and older people after a stroke, found improvements in satisfaction with community reintegration and a significant increase in self-efficacy related to participation [23, 24].

In two qualitative studies, stroke individuals living in the community (three months – 18 years post-stroke) described self-management as an important, unavoidable feature of life after a stroke, but they were not ready and felt ill-prepared to self-manage post-discharge. They missed having professional empowering support post-discharge, and they thought their self-management support needs were often unmet [25, 26].

Furthermore, a Danish study concluded that an inclusive mentor relationship focusing on empowerment might facilitate a changing process. The participants were marginalized people who could regain the loss of the ability to act, be a part of ‘something more’ and be a full member of positive communities of practice [27].

Overall, the above mentioned existing literature has indicated that older stroke individuals might benefit from health professional-led self-management programmes – especially in terms of reintegration and participation in the community, increased quality of life and improved self-efficacy. Furthermore, the existing literature has indicated that stroke individuals enquired about health professional-led self-management support post-discharge.

The objectives of this study are: 1) to describe the underlying social psychological mechanisms and processes that are expected to increase or maintain self-management behaviour from the stroke individuals’, informal caregivers’ and mentors’ perspectives and, 2) to evaluate the feasibility and acceptability of the social psychological mechanisms and processes of the intervention from the same three perspectives.

## Methods

### Design

**Fig. 2:**
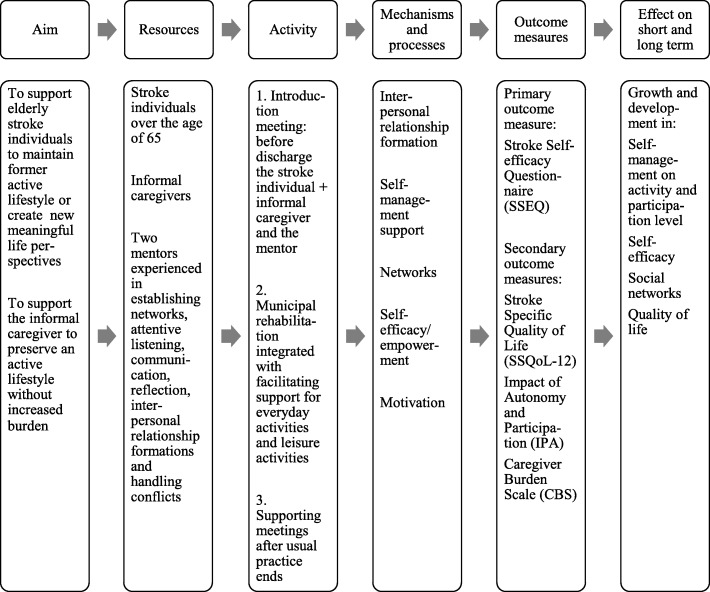
A preliminary simple logic model of the self-management intervention

We used a systematic, stepwise approach, consistent with the MRC Framework for developing and evaluating complex interventions [20]. The intervention was developed based on existing evidence and social cognitive theories. Based on the development phase we described the expected content in the intervention generally. Hereby, we could establish a sound foundation for the evaluation of the mechanisms and processes of the intervention. A simple logic model of the expected content is presented in Fig. 2.

After the development phase, we evaluated a 9 month feasibility test of the intervention including four stroke individuals, their informal caregivers and two mentors by conducting qualitative interviews. A qualitative phenomenological approach was chosen as the purpose of the study is to describe and illuminate the experiences with and perceptions of mechanisms and processes in the intervention, from the informants’ perspectives. A phenomenological approach enables the researcher to understand social phenomena from the informants’ own perspectives and describe the world as experienced by the subjects [28–30].

### Study setting and participants

The intervention evaluated in this study is cross-sectoral. It took place in both a specialized neuro-rehabilitation hospital in a Danish region with 1.2 million inhabitants, and in a specialized neuro-rehabilitation centre in a Danish municipality with 336,000 inhabitants.

Four stroke individuals and their informal caregivers completed the intervention in the feasibility test. Two experienced mentors conducted the intervention. In the evaluation phase, all four stroke individuals, three informal caregivers and the two mentors participated in the qualitative interviews.

The intervention was feasibility tested from March to December 2017. We conducted the interviews with the stroke individuals and their informal caregiver 6–9 months post-discharge, just before the final meeting with the mentor. The two mentors were subsequently interviewed at the end of the feasibility test. The evaluation phase lasted from November 2017 to January 2018.

### Selection of participants

Four stroke individuals over the age over 65 were recruited to participate in the feasibility test. The stroke individuals were selected if they met the following eligibility criteria: 1) were the first willing participant from each of the four participating neurorehabilitation hospitals/centres, 2) had no severe cognitive impairment, defined as < 20 on the Montreal Cognitive Assessment (MoCA), 3) spoke and understood Danish, and 4) were to be discharged to own home. All four stroke individuals agreed to participate and completed the feasibility test. They all had an informal caregiver of their own choosing who took part in the stroke individual’s rehabilitation process.

The four selected stroke individuals and their informal caregivers from the feasibility test were all invited to participate in an interview to evaluate their experiences of undergoing the intervention. Provision was made for up to three informal caregivers to participate. Characteristics of the stroke individuals and their informal caregivers are shown in Table 1. In addition, we interviewed the two mentors, who were the sole conductors of the intervention. Characteristics of the mentors are shown in Table 2.

**Table 1:** Characteristics of the stroke individuals and their informal caregivers

Stroke individual (SI)	Gender	Age	Informal caregiver’s relation to SI (age)
SI 1	Female	65–74	Spouse (65–74)
SI 2	Male	65–74	Cohabitant (Not interviewed)
SI 3	Male	75–84	Son (35–44)
SI 4	Male	75–84	Spouse (75–84)

**Table 2:** Characteristics of the mentors in the self-management intervention

	Basic education	Clinical practice experience	Educational level/Additional training relevant to mentoring
Mentor 1 (M1)	Physiotherapist	> 20 years	Health professional coach trainingCaregiver courses
Mentor 2 (M2)	Occupational therapist	> 20 years	Clinical instructor (occupational students) Diploma in Health, Caregiver courses

### The interviews

We developed two semi-structured interview guides with open-ended questions – one for the stroke individuals and their informal caregiver and one for the mentors, cf. Additional files 1 and 2. The interviews addressed the informants’ experiences, sensations and feelings of either receiving or conducting the intervention. The use of open-ended questions allowed the informants to speak freely and tell about their experiences.

Five interviews were conducted with a total of nine informants; three dyadic interviews with stroke individuals and their informal caregivers, one single interview with a stroke individual and one dyadic interview with the mentors. Dyadic interviews are defined as an interview carried out with two persons at the same time [31]. For pragmatic reasons, the four interviews with the stroke individuals and their informal caregivers were conducted first, followed by the interview with the mentors.

The first and second authors were both present at all interviews – one led the interview and the other asked follow-up questions, when relevant. The interviews with the stroke individuals and the informal caregivers were conducted in the stroke individuals’ homes and lasted approximately one hour. The interview with the mentors was conducted at their workplace and lasted approximately one and half hours. The two first authors audio recorded and transcribed the interviews verbatim in Danish.

### Data analysis

The interviews were analysed with inspiration from the phenomenological approach of Giorgi [28, 29] and Kvale & Brinkmann [30], based on a five-step analytical approach.

*Step 1 – reading and rereading the whole interview in order to gain a sense of the whole.*

Both the first and second authors read the interviews in printed forms and re-listened to the audio recordings to ensure accuracy in the transcriptions.

*Step 2 – natural “meaning units”, as they were expressed by the interviewees, were identified by the researcher.*

The interviews were separately re-read and differentiated into meaning units by the two first authors together. Meaning units were formed each time the authors reached consensus on the occurrence of an instance of transition in the informants*’* experiences of the intervention.

*Step 3 – the dominating themes in the meanings units were identified. The researcher endeavoured to thematize from the interviewed person’s point of view, as the researcher understood it.*

The identified meaning units were transformed into temporary categories and patterns that represented the informants’ natural attitude expressions, as understood by the authors. We did this by manually tracing, identifying and reaching consensus about provisional categories and patterns in the empirical data. NVivo 11.0 was used to organise the traced categories and patterns. In this way, the individual statements were grouped into spontaneously occurring patterns.

*Step 4 – the meaning units were questioned based on the research questions.*

Subsequently, the meaning units were re-read and the informants’ natural attitude expressions were transformed into socially and psychologically sensitive expressions. When we reached consensus about the meaning of the expressions, we then grouped the meaning units into categories that represented the essences of the phenomenon, moving from situated structures to a general structure. The meaning units were moved around until we were convinced that they were all in the right categories. By going back to the raw data and simultaneously condensing meaningful structures, an iterative process occurred and more essential meaning emerged. The most representative and clarified meaning units were then organised.

*Step 5 – condensing the non-redundant themes into descriptive statements.*

**Fig. 3:**
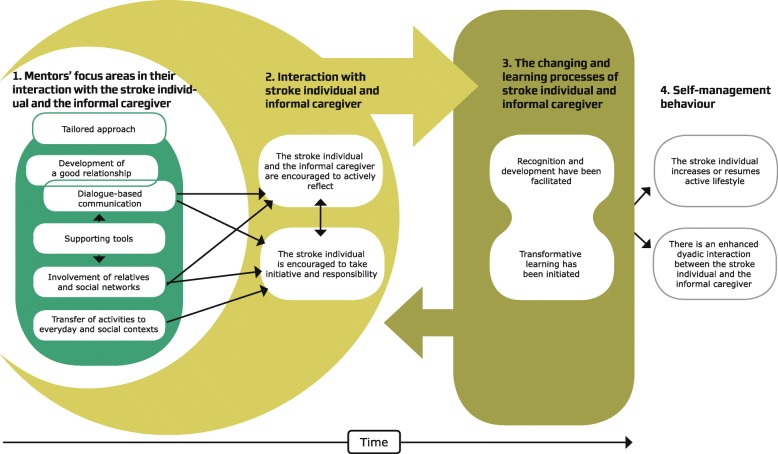
Synthesis of the results

In this final step, the meaning units were synthesised and described in a final set of themes. First, themes were identified in structures as they spontaneously appeared in the data. The two first authors concentrated all the themes together. Thereafter, the themes were shared between the authors, because the variations of the themes were described independently. During the iterative writing process, all three authors continually evaluated and discussed the variations in the themes to ensure credibility, transparency and trustworthiness of the emerging results. Moreover, the themes were adapted until we had an understanding of the underlying social psychological mechanisms and processes that emerged. The insights obtained were synthesised and integrated into consistent statements about the phenomenon. Finally, the connections and variations between the structures were identified, cf. Fig. 3.

The interviews with the stroke individuals and informal caregivers were analysed separately from the interview with the mentors from step 1–4. All interviews were analysed together in step 5. The synthesis of the results were presented to the mentors and the steering committee of the project *‘Stroke - 65 plus. Continued Active Life’* for confirmation.

## Results

The results are presented in the following. Anonymizing codes are placed in parentheses after the quote i.e. (SI1, M1) to indicate the individual informant. In this study, the informants were divided into three groups: the stroke individuals, the informal caregivers and the mentors. From here on, stroke individuals and informal caregivers are referred to as the two parties when they agree on experiences.

The analysis of all the interviews contributed to a comprehensive understanding of the experienced mechanisms and processes in the intervention. The analysis showed that a fundamental condition in the intervention was that the mentors considered the stroke individual and the informal caregiver to constitute both a dyadic unit as well as two individuals. Hereby, the informal caregiver became an active co-participant.

The main results of the analysis were six social psychological mechanisms related to the mentors’ focus areas in their interaction with stroke individuals and informal caregivers: a) Tailored approach – by individual preferences, b) Dialogue-based communication, c) Development of a good relationship, d) Transfer of activities to everyday and social contexts, e) Involvement of relatives and social networks, and f) Supporting tools – to optimise actions and communication. These mechanisms were described by the informants to be vital in supporting self-management behaviour. By way of the mentors’ focus areas, the stroke individuals and their informal caregivers were encouraged to actively reflect and to take initiative and responsibility. Hereby, the stroke individuals and their informal caregivers became participants in the actions, and important interaction processes between the mentor, stroke individual and informal caregiver occurred. The active ingredients in the interaction process could generate processes of change and learning, as the stroke individuals and their informal caregivers attained new perspectives in relation to reintegration into the community. The new learning could lead to a change in the stroke individual’s and the informal caregiver’s perception of their self-identity. The above-mentioned social psychological mechanisms and processes seemed to increase self-management behaviour, in terms of an active lifestyle. Furthermore, the stroke individuals and their informal caregivers experienced an enhanced dyadic interaction between them.

The synthesis of the results is illustrated in Fig. 3 and will be further elaborated in the descriptions that follow.

### Step 1: social psychological mechanisms

The six revealed social psychological mechanisms formed the basis of the mentors’ focus areas in the intervention. A mechanism is here understood as a psychological factor or a system of factors that influences a stroke individual’s or informal caregiver’s behaviour. The six mechanisms will be presented separately in the following, and are listed in Additional file 3.

#### 1a: tailored approach – by individual preferences

The mentors worked with a wide variety of stroke individuals, some of whom were able to handle their problems on their own with a little support, while others needed more support. Based on this, the mentors’ fundamental philosophy when interacting with stroke individuals and their informal caregivers was to have an individualised approach that was flexible and responsive to individual needs and preferences.

The mentors tailored their approach based on knowledge gained from the other mechanisms, which will be presented later in this article. The introduction meeting, cf. Fig. 2, with the stroke individual at the discharging hospital was emphasized by the mentors to be very important. The mentors stated that the benefits of the introduction meeting were to get to know the stroke individual’s and the informal caregiver’s needs and preferences. Furthermore, the mentors gained an insight into which approach would be the most relevant to each individual case based on the self-understanding, roles and habits expressed by the two parties. Mentor 2 explained the value of the introduction meeting:

*“We (the mentors) try to work out what kind of person it is that we’re going to meet at their home, and we prepare ourselves mentally in relation to what we’re going to encounter when we go there” (M2).*

Mentor 1 elaborated how the valuable knowledge gained at the introduction meeting led them to adapt their approach:

*“So, we knew that, in any case, it wasn’t that (prescriptive) approach that we should take. We would have to find another way, if we were going to reach (the stroke individual) at all” (M1).*

The mentors emphasized that the introduction meeting allowed them to be well prepared to meet and support the stroke individual and informal caregiver post-discharge, taking a tailored approach. Although the introduction meeting seemed to be vital to the mentors, the stroke individuals hardly recalled it and they did not emphasize it as especially important.

The two parties described that the mentors based their support on their needs and preferences. One of the stroke individuals explained how he experienced that the mentor took his needs and preferences into account in the rehabilitation process:

*“There were a lot of questions from her (the mentor) to me about what I needed and what I felt should happen” (SI 2).*

The two parties also stated that the mentors involved and supported them to make shared decisions and that the mentors respected their autonomy. For instance, the mentor encouraged one of the stroke individuals, who pre-stroke had work meetings around the country, to organise a train ride, as it was very meaningful and important to him. The mentor and the stroke individual afterwards took the train ride to make sure he could manage it and hereby, helping the stroke individual to regain his autonomy.

The mentors articulated that the intervention also took a tailored approach on the organizational level, because of the introduction meeting as well as the supporting meetings, cf. Fig. 2. In usual practice, there would be about 14 days’ gap in the rehabilitation process in the transition period post-discharge, before the stroke individual would be contacted by the Visitation unit. In this study, the organisation was tailored so that the stroke individuals did not wait for their rehabilitation plan to be processed by the Visitation unit. The mentors stated that, because of the study set-up, with the introduction meeting, the stroke individuals were able to continue their rehabilitation immediately after discharge. The mentors emphasized this as a way to ensure a sense of security in the transition from hospital to home. Because of this organizational change, the mentors were able to adapt their approach from the beginning. They explained that they had a dialogue with the stroke individual in regard to whether s/he wanted to start the municipal rehabilitation on the day after discharge or preferred to be at home for a couple of days before continuing rehabilitation.

Neither of the two parties articulated uncertainty in the transition from discharge to own home. One of the stroke individuals related that he had been worried about how to manage on his own at home, but because of the support he had from both home-care staff and the mentor, it never became a problem for him. The two parties experienced good coherence in their rehabilitation processes and that the process was adjusted individually. None of them addressed unmet needs after municipal rehabilitation ended in the phase where the mentors continued with supporting meetings. Some of them sought to stay in contact with the mentor after the intervention terminated, as the spouse in case 4 explained:

*“... all that help that was there in the beginning, it just flakes away, ... then there’s us two left in the end ... It would be really good if, just once in a while, a mentor came by ... New issues arise along the way, and there isn’t anyone else” (Spouse, SI 4).*

The mechanism *‘Tailored approach’* was revealed in the results as the mentors’ fundamental philosophy in the interaction with the stroke individuals and their informal caregivers.

#### 1b: dialogue-based communication

Communication was emphasized by the mentors as the most central factor in the provision of the mentor function in general.

The focus was on dialogical conversation and the questioning technique was open and facilitative. Mentor 2 explained it like this:

*“To get asked about (...) things, so (as mentors) we don’t just go out to (the citizen and the relative) with some preconception or other … What do they actually want?, What is important to them?” (M2).*

Mentor 1 continued explaining about the questioning technique:

*“... it’s about the open questions that you (as a mentor) ask, it’s about (enquiring about) their everyday lives, and their wishes for their lives…” (M1).*

The two parties experienced the communication methods positively and they found that the conversations were dialogical. They considered that they were involved in decision making through the intervention, as they could make decisive decisions when they wished, but also found it supportive that the mentor sometimes took the lead.

Through communication, they received psychological support and felt supported to increase their self-efficacy. Two of the stroke individuals expressed it in the following ways:

*“... we ended up having some really good chats, and mentally (the mentor) is also a good support, in my opinion” (SI 4).*

*“...(the mentor) has been a good support to move forward with things ... It (has more been) a question of getting back-up to do (things) … like psychological support … in that way it’s been really good” (SI 2).*

The results revealed that the mechanism *‘Dialogue-based communication’,* by way of dialogical conversations, supported increased self-efficacy in the stroke individuals and caregivers. *‘Dialogue-based communication’* and the linked focus area *‘Development of a good relationship’* were emphasized by the mentors as very central mechanisms in initiating the interaction process between all three parties, where the aim was to encourage active reflection and take initiative and responsibility, and thereby be able to self-manage the new life situation. The interaction processes will be elaborated later in this article.

#### 1c: development of a good relationship

The mentors stated that a focus on a good interaction was essential if the collaboration between the three parties was to be successful. They specified that the interaction between the three parties was different from what they had previously experienced in their usual practice. Mentor 2 explained:

*“The relationship we have with the citizens here is something special ... something else, (something interpersonal) happens in the relationship” (M2).*

As examples of the differences in this intervention compared to usual practice, the mentors described that the stroke individuals addressed other subjects, were more open and asked different questions – for instance, about their sex life. The mentors argued that their relationships with the stroke individuals and their informal caregivers differed in this intervention compared to usual practice because of the longer time horizon in the intervention; as Mentor 2 stated:

*“(As a mentor) you are more patient and open to thinking that the topics (the stroke individual) comes up with are ok … Because they also mean something for (the stroke individual) in the bigger picture” (M2).*

The mentors explained that they sought to develop a good relationship by focusing on being present in the moment, listening attentively, being open-minded and by their communication methods.

The two parties all described the collaboration with the mentors as positive. They emphasized the mentors’ personality characteristics as being authentic, empathetic, having flair, attentive listening skills, being thorough and consistently following up on agreements and encouraging; and that these were important for them to trust and collaborate with the mentor.

An informal caregiver highlighted the following as important characteristics of a mentor:

*“... (the mentoring) has been a really good experience. As I said, I started out being sceptical, and that scepticism is gone … (the mentor’s) way of being. And her personality. She is such a warm and friendly person … and has an awful lot of empathy …” (Spouse, SI 4).*

Furthermore, the two parties emphasized that it was important to them that the mentor had neurological knowledge and experience of working with the target group. The mentors were able to inform them about what could be expected in such a rehabilitation situation, thereby normalizing the situation.

The two parties experienced that, together with the mentor, they had developed a good relationship. One informal caregiver expressed it in the following statement to a stroke individual:

*“(The mentor has) been good at making contact. (The mentor is) very straightforward (and) I think (the mentor) has had a good relationship with you ... You have opened yourself up right from the start and have felt secure ... We can (only) say really good things about the (mentoring)” (Spouse, SI 1).*

Another informal caregiver addressed this to the stroke individual:

*“I’m sure that (the mentor) has made a difference by winning your trust” (Son, SI 3).*

In the results, the mechanism *‘Development of a good relationship’* was revealed as being closely related to and integrated into the mechanism *‘Dialogue-based communication’*.

#### 1d: transfer of activities to everyday and social contexts

The focus on getting the stroke individuals to participate in their local community as quickly as possible following discharge from hospital was emphasised as important by the mentors.

The mentors were aware throughout the intervention that training in the stroke individual’s home or social context increased the ability to transfer the learning to everyday life. Mentor 2 explained it like this:

*“Everything that they (the stroke individuals) train must be brought across to their everyday lives. That transfer element is implicit for us (the mentors)” (M2).*

Mentor 1 supported the above point of view:

*“That point about being very aware of .... Should (the stroke individual) come in here to train at all, or should they train at home … I have become more aware when it is that we (the mentors) bring them in here … (and) when they should stay out (and train in their own environment)” (M1).*

One of the informal caregivers described that his father had resumed a more active lifestyle, because the mentors had trained with and encouraged him in his own context:

*“The options that they offered … have made a huge difference. You (referring to his father) had become much more passive (before the offer) … I, in any case, am completely convinced that it has been a very positive contribution to the fact that my father can now walk by himself down to the Concert Hall” (son, SI 3).*

In addition, the mentors emphasized that training performed in the stroke individuals’ homes or social contexts increased the opportunity to focus on their most meaningful and enjoyable activities. This could be shopping at the local grocery store, making pancakes and apple pie in their own kitchen, travelling by train, and so forth. Furthermore, the mentors used nature to promote the joy of doing the activities, for instance, collecting autumn leaves and putting them on a string in order to train the upper extremity. Another example was cooking over a fire, which was described by one of the stroke individuals:

*“There were a couple of us who … did a lot of fun things in there (in the rehabilitation centre) with the mentor; for example, (we) … cooked over a fire … That gives you a lot of training … And it was fun” (SI 2).*

According to the results, the focus on the mechanism *‘Transfer of activities to everyday and social contexts’* enabled the stroke individuals to take initiative and responsibility, because it was easier for them to cope with everyday life when they had been supported in their own context.

#### 1e: involvement of relatives and social networks

The mentors pointed out the importance of involving relatives and social or other networks, to reduce dependence on mentor support and to prevent loneliness and marginalization.

They found variance in how much the informal caregivers wanted to be involved in the rehabilitation process, so they made adjustments accordingly. All the informal caregivers expressed that it was beneficial for them to be involved in the rehabilitation process. This was expressed in the following:

*“... being involved in the process is important ... to get the right picture of what my father has been through ... and to be able to support him” (Son, SI 3).*

With enthusiasm, the informal caregivers also described themselves as co-mentors and co-motivators in the rehabilitation process. They all felt well prepared by the mentors to engage in the co-operative role, and the support from their informal caregivers made the stroke individuals feel very secure. During one of the interviews, a stroke individual realized the importance of her husband’s support, and described it like this:

*“... I hadn’t ever thought about the fact that you (referring to her husband) are actually the mentor’s extended arm … because you are the person who backs me up day to day” (SI 1).*

However, in one single case, the informal caregiver was described by the mentors to be a limiting factor in the rehabilitation process:

*“.... she is the type who just does things lightning fast, and it’s been a big issue throughout (the stroke individual’s) rehabilitation ... to get her to see that her servicing simply has to stop, because otherwise (the stroke individual) won’t develop himself” (M2).*

In several cases, the mentors described themselves as marriage mediators; they had a significant role in encouraging mutual understanding, e.g., by ensuring that the stroke individual and informal caregiver both listened to each other’s perspectives on a given issue. This was supported by a description from one of the spouses:

*“... one way or another, the conversations with the mentor have been really good. As (my husband) says, the mentor has been good at putting words on some things between him and I … It is very hard to say anything specific, but the mentor has been a good mediator (concerning the issues that crop up along the way after a stroke)” (Spouse, SI 4).*

Furthermore, one of the stroke individuals described it this way:

*“... I preferred to do the kitchen work myself, which gradually became annoying for my partner … So we had a chat with the mentor about it … There’s no doubt that it was becoming a conflict point … so it was good that the mentor sorted it out” (SI 2).*

Relatives and social networks were mentioned by the two parties, but they did not express the need for further help from the mentors in relation to their involvement.

The results revealed that the mechanism *‘Involvement of relatives and social networks’* contributed to active reflection and to the stroke individuals taking initiative and responsibility.

#### 1f: supporting tools – to optimise actions and communication

The mechanism ‘*Supporting tools’* was made up of a non-technological and a technological element. The non-technological element was used the most. The mentors used the two non-technological supporting tools ‘network card’ and ‘areas of importance’ consistently. Their Social networks were mapped by using the ‘network card’. The ‘areas of importance’ were used to give a meaningful picture of what was most important to the stroke individual. The two tools gave the mentors a valuable reference framework for their supportive actions throughout the entire rehabilitation process. The two parties did not recall the supporting tools as being significant. Furthermore, the mentors mentioned a logbook (mainly to activate reflection) and a ‘mentor book’ (mainly to note agreements) as additional non-technological supporting tools that would be used ad hoc.

A paper calendar emerged as a significant non-technological supporting tool for the stroke individuals. They used a calendar pre-stroke, but were more dependent on it post-stroke, as it helped them to create structure in their everyday lives and to support their memory. They only needed minimal support from the mentors regarding the use of the paper calendar.

The technological supporting tool consisted of an app called the ‘Life-Manager’, which could be downloaded to the stroke individual’s own smartphone or iPad. If necessary, an iPad could be borrowed for the purpose. The app was the stroke individual’s own digital ‘book’, which could be used to optimize planning and communication with informal caregivers and mentors. All three parties provided negative feedback on the app. One stroke individual described it like this:

*“... I certainly don’t think much of Life-Manager … There are some things that don’t work … It doesn’t do at all” (SI 3).*

The criticism concerned technical issues and not least the lack of meaningfulness. The mentors agreed that the app did not add extra value as a supporting tool.

The mentors recommended that the technological issues regarding Life-Manager be resolved and that increased attention be placed on the introduction to the app. If Life-Manager were not to be improved, the mentors recommended that it should be omitted from the intervention as a supporting tool.

The mentors stated that the social psychological mechanism *‘Supporting tools’* supported their communication and could give an overview of which relatives and networks should be involved.

### Steps 2 and 3: social psychological processes

The social psychological processes revealed seem to be initiated and facilitated through the social psychological mechanisms – the mentor’s focus areas, and were defined as a sequence or series of acts that involved change and development within the stroke individuals and possibly also the informal caregiver. The processes consisted of the interaction process, which was divided into active reflection and taking initiative, plus the changing and learning processes. The processes will be presented in the following.

#### 2a: active reflection in the interaction process

The mentors facilitated the stroke individuals and their informal caregivers to actively reflect, especially through the dialogical conversations. In this interaction process, the mentors’ focus was on facilitating initiative and active reflection. As mentioned earlier, the mentors used communication methods to facilitate reflection. Together, all three parties agreed on and, e.g., wrote down actions to be performed or initiated before the next meeting. At the following meeting, they followed up on the agreements. The mentors initiated a reflection process by asking questions like: What did it mean to you to go out and teach again? Why don’t you go down to the Concert Hall? What has to happen for you to get out and about again? This implied that the mentors gave space so that, through specific experiences, the two parties could reflect on their own actions, self-management and self-identity. Mentor 1 explained the mentor’s role in initiating active reflection:

*“... we have to be the ones who try to give space to ... thoughts and experiences, and so he (the citizen) has to reflect further on it himself … we can’t fix anything” (M1).*

The two parties experienced that the dialogical conversations with the mentors as a third neutral participant facilitated new perspectives on their situation and enabled them to use these perspectives to reflect and find new solutions or acceptance. One informal caregiver stated:

*“(The mentor) is good at just dropping in those key words so that things come out ... That’s our (the stroke individual and wife) starting point, that we get talking about these things and anyway it’s good that (the mentor) is here … So we get to express all these misunderstandings that can’t be avoided at home” (Spouse, SI 4).*

In analysing these processes, it appeared that both the stroke individual and informal caregiver underwent active reflection, learning and change. The active reflection aimed to increase awareness and thoughts regarding reintegration into the community.

#### 2b: to take initiative and responsibility in the interaction process

The mentors emphasized the importance of the stroke individuals taking initiative and responsibility, so they would be able to conduct an active life on their own after the intervention ended. Therefore, the mentors were aware of getting the stroke individuals to be active themselves, as expressed in the following quote:

*“I have become even more acutely aware ... of getting them (the stroke individuals) to act themselves, instead of us (the mentors) taking over” (M2).*

Later in the interview, this was further exemplified:

*“For example, (one of the stroke individuals) was to try to find the programme for the Concert Hall … because he wanted to go over there more … So, it was his task for next time … that he was to find the programme and have a go at going over there” (M1).*

In encouraging the stroke individuals to take initiative and responsibility, it amounted to empowerment. The mentors facilitated empowerment by highlighting various possibilities in order to encourage the stroke individuals discover and orient themselves towards the most meaningful and enjoyable activities in their lives.

The two parties experienced that it was beneficial to make agreements in co-operation with the mentors in relation to resuming previously experienced meaningful and enjoyable activities. One of the stroke individuals explained that a big part of his life pre-stroke was to give lectures and courses. He was very dependent on his speech, which was affected post-stroke. The stroke individual and the mentor therefore made the following agreement:

*“The mentor and I agreed that it was a good idea to encourage people to ring me, because it forced me to talk” (SI 2).*

Another stroke individual also explained how he was encouraged by the mentor to do something by himself:

*“The mentor has encouraged me to make a web page ... about my trip over to the Concert Hall, but there are other (web pages) that I have hooked up with” (SI 3).*

The mentors used encouragement and supported initiative and responsibility as an intentional strategy. They found it to be a successful method to empower self-management in the new life situation. The initiative and responsibility were directed towards concrete actions focusing on behaviour change with the intention of augmenting an active lifestyle.

### 3: The changing and learning processes

The active ingredients in the interaction process were found to generate processes of change and learning in the stroke individual and their informal caregivers. This could be increased knowledge and understanding of what to expect post-stroke or an emotional development, for instance increased self-efficacy.

Furthermore, a transformative change was facilitated as the perception of self-identity changed during the rehabilitation process. The mentors experienced that the stroke individuals’ self-identity did not necessarily change at the same pace as the development of an active lifestyle. This was exemplified in the following quote:

*“… We (the mentors) simply thought that it had been going so well (for one of the stroke individuals) … when we suddenly find out … He has not changed his self-image very much … He still considers himself really to be a twerp” (M1).*

Through critical self-reflection initiated by the mentor, subsequently the stroke individual transformed how he thought of himself and his life:

*“I’m happy about it (the collaboration with the mentor) ... We got to talk about some of the things that I find annoying … I have been irritated with (myself). (I have felt myself to be) a twerp … (The mentor) has been good at building me up again” (SI 2).*

### Step 4: self-management behaviour

This study has shown that the identified social psychological mechanisms and processes seemed to increase or maintain self-management behaviour in two ways: 1) The stroke individual increased or resumed an active lifestyle. Although the activities were not resumed to the same level as pre-stroke, this was not crucial to the stroke individuals, because it was the meaningfulness of the activities that counted. 2) The relationship between the stroke individual and the informal caregiver was an enhanced dyadic interaction. This applied especially to their mutual understanding. These actions were regarded as contributions to managing their lives post-stroke, to maintain the best possible quality of life.

## Discussion

All the informants – the stroke individuals aged over 65, the informal caregivers and the mentors – expressed that the novel health professional-led self-management support intervention was meaningful and relevant, with the exception of the technological supporting tools. The major results in this study were that the informants experienced that the social psychological mechanisms and processes were relevant to their situations and that the mentors contributed with helpful social psychological support throughout the entire intervention. Furthermore, the mentors perceived the stroke individual and the informal caregiver both as a dyadic unit, and as two individual persons.

It is recommended that consideration be given to how the technology part could be made meaningful and supplement existing technology, which is why further work is needed to develop and refine this part of the intervention. The technological supporting tool is not considered to be decisive to the outcome of the novel self-management support intervention. The social psychological mechanisms and processes were perceived to be feasible and acceptable to the stroke individuals, the informal caregivers and the mentors. Therefore, apart from the technology, the social psychological mechanisms and processes are considered to be implementable into the RCT in the larger project *‘Stroke - 65 plus. Continued Active Life’*.

Previous research has identified that stroke individuals and their closest relatives experience uncertainty when they go from one phase to another, e.g., the transition from hospital to home or after completion of municipal rehabilitation [32]. None of the informants in this study described any uncertainty in this regard. The absence of uncertainty seemed to be due to the mentors’ ‘pulling’ philosophy [32]; the mentors were aware of actively ensuring that encouraging and facilitating follow-up sessions occurred throughout the entire intervention period.

It is well known that stroke individuals often suffer from cognitive sequelae post-stroke [33, 34]. Cognitive difficulties can be expressed in terms of reduced initiative, difficulties in active reflection and memory impairment [35]. This was taken into account in the design of the study, by involving the relatives and social networks in the rehabilitation process. The mentors took part in facilitating that the stroke individuals could get support in everyday life which could help them to take appropriate responsibility and develop their independence adapted to their ability.

Self-identity can be affected in numerous ways post-stoke, and the stroke individuals seemed to redefine their self-identity by way of all their interactive situations [36]. All the stroke individuals participating in this study were involved in a process in which they redefined themselves regarding their reintegration into the community, and they were supported by the mentors to undergo this transition. This is similar to a study with young stroke individuals, where support from health professionals is mentioned as a contributing factor that facilitated transformative learning [37]. The intervention in the present study contains all the core elements that must be present to initiate transformative learning, and it seems to be an important part of the intervention to increase or maintain self-management behaviour among older stroke individuals over the age of 65.

There is increased focus on self-management support interventions for stroke individuals, but further research on the key features of self-management programmes and how to deliver them is required [21, 22]. Jones has suggested the use of the ‘Bridges stroke self-management programme’ [38–40], which integrates an individualized approach based on self-efficacy principles into stroke rehabilitation as a method to improve long-term condition for stroke individuals.

The mechanisms and processes discovered to support stroke individuals in this study coincide in some aspects with the components in the ‘Bridges stroke self-management programme’. Both programmes are underpinned by social cognitive theory and focus on supporting self-efficacy [38, 40, 41]. Compared to the ‘Bridges stroke self-management programme’, there is a more systemic approach in this current study that regards the stroke individual and informal caregiver as a unit, rather than two separate individuals. Both the ‘Bridges stroke self-management programme’ and the intervention in this study focus on facilitating the stroke individual and the informal caregiver to actively reflect and to take initiative and responsibility on the activity level [39]. However, this study contributes by also facilitating active reflection and the taking of initiative and responsibility on the participation level – by involving social networks and social context. Furthermore, the length of the intervention in the current study allowed for changing and learning processes to occur. The length of time was an important prerequisite for change and transformation. This novel self-management intervention was experienced by the stroke individuals and informal caregivers as having a positive effect in the rehabilitation process.

### Strengths and limitations of the study

To our knowledge, this is the first study to focus on resuming an active lifestyle and enhancing dyadic interaction between older stroke individuals aged over 65 and their informal caregivers.

The strength of this study lies in the in-depth exploration of experiences of the underlying social psychological mechanisms and processes in the intervention, from the perspectives of stroke individuals, informal caregivers and mentors. This was obtained by the application of a qualitative phenomenological approach. The synthesis of the results was confirmed with the mentors, to ensure validity of the results.

The study was inspired by the MRC guidance, which recommends a combination of quantitative and qualitative methods [20, 42]. The current study takes only a qualitative approach. The quantitative measures, such as the number of meetings, the length of the intervention and so on, could have contributed to a more complete picture of the informants’ experiences of the intervention. However, the absence of the quantitative measures is not considered to affect the qualitative results in this study.

It is a limitation of this study that only four stroke individuals, all of whom had an informal caregivers and two mentors were included. In this setting, an informal caregiver is a person living with or separately from the stroke individual. They had to have a significant personal relationship with, and provide a broad range of assistance to the stroke individual during the rehabilitation period. The results from this study are, therefore, not generalizable for stroke individuals without informal caregivers. The results do, however, indicate the importance of involving an informal caregiver in the rehabilitation process. Furthermore, the results indicate the vulnerability among those stroke individuals who have no informal caregiver and are completely dependent on their own inner resources and support from health professionals.

It is also acknowledged that this study included only two mentors, both of whom worked at the same municipal rehabilitation centre. If the intervention were to be implemented in a different municipality, different results might arise. In that case, the intervention must be adapted to the given context.

A limitation in self-management research in general is the lack of consensus about what elements should be present in a self-management intervention [15, 21, 40]. Despite the limitations of the study design, this study provides detailed descriptions of the social psychological mechanisms and processes in an integrated self-management intervention targeting behavioural change and self-efficacy.

### Study implications

The current study was designed using the MRC guidance that recommends evaluating the feasibility and acceptability of a complex intervention before implementing the intervention into an RCT [19]. The results of this study on the feasibility and acceptability of the intervention from the informants’ perspectives will be implemented into the RCT in the larger project *‘Stroke - 65 plus. Continued Active Life’.*

Furthermore, the results of this study are important and useful for the intervention to be implemented in other contexts and to inform the future design and development of self-management research.

## Conclusion

The analysis of the data revealed six social psychological mechanisms in the self-management intervention related to the mentors’ focus areas in their interaction with stroke individuals and informal caregivers: a) Tailored approach – by individual preferences, b) Dialogue-based communication, c) Development of a good relationship, d) Transfer of activities to everyday and social contexts, e) Involvement of relatives and social networks, and f) Supporting tools – to optimise actions and communication. The mechanisms were associated with interaction processes between the mentor, stroke individual and informal caregiver. Furthermore, processes of change and learning occurred in the stroke individuals and their informal caregivers. The mechanisms and processes indicated a positive association to self-management behaviour from the informants’ perspectives and were perceived as feasible and acceptable to the informants – with the exception of the technological supporting tool.

## Additional files

Additional file 1: Interview guide - mentor: Translation of Danish interview guide (DOCX 19 kb)

Additional file 2: Interview guide – stroke individual and relative: Translation of Danish interview guide (DOCX 19 kb)

Additional file 3: **Appendix.** Operationalization of the six social psychological mechanisms - the mentors’ focus areas – in the self-management intervention (DOCX 16 kb)

## Abbreviations

CBS: Caregiver Burden Scale
COPM: Canadian Occupational Performance Measure
IPA: Impact on Participation and Autonomy
MoCA: Montreal Cognitive assessment
SSEQ: Stroke Self-efficacy questionnaire
SSQoL: Stroke Specific Quality of Life Scale
UK MRC: United Kingdom Medical Research Council
