# Supplementary material for: Social psychological mechanisms and processes in a novel, health professional-led, self-management intervention for older stroke individuals: a synthesis and phenomenological study
Source: BMC Health Serv Res. 2019 May 22;19:320. doi: 10.1186/s12913-019-4150-x (PMC6530065; doi:10.1186/s12913-019-4150-x)
Supplement: Supplementary file 1 — Interview guide - mentor: Translation of Danish interview guide (DOCX 19 kb) [file 12913_2019_4150_MOESM1_ESM.docx]

*Interview guide – stroke individual and relative.Translation of Danish interview guide*

| Main categories | Questions |
| --- | --- |
| Significant elements in the mentor intervention concerning managing everyday life | Can you tell us about your experience of your first meeting with the mentor before discharge?  Did you and your mentor work with how you could manage your everyday life on your own?  Did the mentor try to involve your family and friends in your rehabilitation?  Did the mentor support you to be a part of social networks?  Have you been involved in forming and deciding your rehabilitation?  Would you say that the mentor intervention has made a difference to your way of managing your life situation? |
| The relevance and quality of the mentor intervention | How did you experience having a mentor?  Is it important that the mentor is a professional?  Can you imagine how your life would have been without the support?  Do you have suggestions about how to make the support better? |
| The significance of the supporting tools in the rehabilitation process | Which supporting tools have you used?  How have you used the supporting tools?  How often have you used them?  Did the supporting tools made any difference to your everyday life? |
| The significance of the support from the mentor to the relative | How did you experience the interaction with the mentor?  Did the mentor support you? |
| Conditions for interaction with the mentor | How often have you been in contact with the mentor?  Where did the contact take place?  Did anything impact your chance to meet with the mentor? |
