# Supplementary material for: Social psychological mechanisms and processes in a novel, health professional-led, self-management intervention for older stroke individuals: a synthesis and phenomenological study
Source: BMC Health Serv Res. 2019 May 22;19:320. doi: 10.1186/s12913-019-4150-x (PMC6530065; doi:10.1186/s12913-019-4150-x)
Supplement: Supplementary file 2 — Interview guide – stroke individual and relative: Translation of Danish interview guide (DOCX 19 kb) [file 12913_2019_4150_MOESM2_ESM.docx]

*Interview guide – mentor. Translation of Danish interview guide*

| Main categories | Questions |
| --- | --- |
| General experience of mentoring | Can you tell us about your experience of the first meeting with the stroke individual and relative before discharge?  What are your experiences of being a mentor in the phase involving municipal rehabilitation?  How did you experience the phase involving the supporting meetings? |
| Experiences of supporting the stroke individuals to increase or resume an active lifestyle | How did you experience supporting the stroke individuals to resume an active lifestyle? |
| What are the important social psychological elements in the mentor intervention? | Based on your experiences, what are the most important elements of the mentor intervention?  Did you work towards the active involvement of the stroke individual?  To what extent did your expectations of involving the relatives meet with the reality in practice  How did you experience involving the stroke individuals' networks in the rehabilitation?  What, in your opinion, is the significance of the mentor intervention to the stroke individual and the relative? |
| How the mentor intervention differs from usual practice | Can you describe how the phase involving municipal rehabilitation differed from usual practice? |
| The significance of the supporting tools | Will you describe how it has been to use the supporting tools?  Did you find the supporting tools relevant and useful? |
| Conditions for interaction with the stroke individual and relative | How many meetings have you had with the stroke individual and relative?  When and where did the meetings take place, and how has the contact been?  Did anything impact on your chance to meet with the stroke individuals and relatives? |
