# Supplementary material for: Social psychological mechanisms and processes in a novel, health professional-led, self-management intervention for older stroke individuals: a synthesis and phenomenological study
Source: BMC Health Serv Res. 2019 May 22;19:320. doi: 10.1186/s12913-019-4150-x (PMC6530065; doi:10.1186/s12913-019-4150-x)
Supplement: Supplementary file 3 — Appendix. Operationalization of the six social psychological mechanisms - the mentors’ focus areas – in the self-management intervention (DOCX 16 kb) [file 12913_2019_4150_MOESM3_ESM.docx]

*Appendix: Operationalization of the six social psychological mechanisms - the mentors’ focus areas – in the self-management intervention*

| **The mentors’ focus areas** | **How the mentors’ approach is operationalized in practice** |
| --- | --- |
| **Tailored approach**  The mentors adjust their actions for each stroke individual’s and caregiver’s needs and preferences | The mentors seek to tailor their approach to the stroke individuals and their informal caregivers throughout the entire intervention. Their approach is tailored based on knowledge gain from the five other mechanisms. Hereby, the mentors adjust the time, place and the content of the intervention to the needs and preferences of the stroke individuals and their informal caregivers. |
| **Dialogue-based communication**  The mentors seek to have dialogical conversations | The mentors ask open and curious questions and give the stroke individuals and informal caregivers the opportunity to put their current concerns and thoughts into words, e.g., *“What’s on your mind right now?”*, *“What could make it easier for you?”*, *“How would you like it to happen?”*, *“How can we support you?”* or *“What puts a sparkle in your eye and joy in your everyday life?”* |
| **Development of a good relationship**  The mentors meet and acknowledge the resources the stroke individuals and their informal caregiver possess | The mentors give the stroke individuals and informal caregivers time and space to tell their life stories, so they can get insight into and identify their roles and habits. The mentor’s personality, e.g., being authentic, having flair and being empathic, plus their neurological knowledge and experience, are central to developing a good relationship. |
| **Transfer of activities to everyday and social contexts**  The mentors focus on how and if the stroke individuals can transfer the learning to their everyday lives | The mentors support the stroke individuals, both at home and in other relevant contexts, e.g., they replace doing exercises at the municipal’s premises with exercises in the individual’s community, if it makes sense to do so. |
| **Involvement of relatives and social networks**  The mentors involve the stroke individual’s relatives and social networks, based on the stroke individual’s wishes | The mentors will inform and prepare the relatives and people from the social networks of what to expect, so they are prepared to support the stroke individual. Furthermore, the mentors will introduce the stroke individuals to their support options, and whom they can contact, if necessary. |
| **Supporting tools**  The mentors use visual instruments to make the spoken and written language more tangible, to make agreements more binding | The mentors are mapping the social network by using a ‘network card’ and identify the ‘areas of importance’ which could give a meaningful picture of what was most important to the single stroke individual. Furthermore, the logbook, paper calendar and the app ‘Life-Manager’ are used ad hoc. |
